# Balancing Speed and Accuracy in Cardiac Magnetic Resonance Function Post-Processing: Comparing 2 Levels of Automation in 3 Vendors to Manual Assessment

**DOI:** 10.3390/diagnostics11101758

**Published:** 2021-09-24

**Authors:** Gert J.H. Snel, Sharon Poort, Birgitta K. Velthuis, Vincent M. van Deursen, Christopher T. Nguyen, David Sosnovik, Rudi A.J.O. Dierckx, Riemer H.J.A. Slart, Ronald J.H. Borra, Niek H.J. Prakken

**Affiliations:** 1 Medical Imaging Center, Department of Radiology, University Medical Center Groningen, University of Groningen, Hanzeplein 1, 9713 GZ Groningen, The Netherlands; sharonpoort87@gmail.com (S.P.); r.j.h.borra@rug.nl (R.J.H.B.); n.h.j.prakken@umcg.nl (N.H.J.P.); 2 Department of Radiology, University Medical Center Utrecht, University of Utrecht, Heidelberglaan 100, 3584 CX Utrecht, The Netherlands; B.K.Velthuis@umcutrecht.nl; 3 Department of Cardiology, University Medical Center Groningen, University of Groningen, Hanzeplein 1, 9713 GZ Groningen, The Netherlands; v.m.van.deursen@umcg.nl; 4 Department of Radiology, Athinoula A. Martinos Center for Biomedical Imaging, Massachusetts General Hospital, Harvard Medical School, 149 13th Street, Charlestown, MA 02129, USA; Christopher.Nguyen@mgh.harvard.edu (C.T.N.); dsosnovik@mgh.harvard.edu (D.S.); 5 Cardiovascular Research Center, Massachusetts General Hospital, Harvard Medical School, 149 13th Street, Charlestown, MA 02129, USA; 6 Division of Health Sciences and Technology, Harvard-MIT, 7 Massachusetts Avenue, Cambridge, MA 02139, USA; 7 Medical Imaging Center, Department of Nuclear Medicine and Molecular Imaging, University Medical Center Groningen, University of Groningen, Hanzeplein 1, 9713 GZ Groningen, The Netherlands; r.a.dierckx@umcg.nl (R.A.J.O.D.); r.h.j.a.slart@umcg.nl (R.H.J.A.S.); 8 Faculty of Science and Technology, Department of Biomedical Photonic Imaging, University of Twente, Dienstweg 1, 7522 ND Enschede, The Netherlands

**Keywords:** cardiac magnetic resonance, cardiac function, reproducibility, automation, standardization, tracing protocol

## Abstract

Automating cardiac function assessment on cardiac magnetic resonance short-axis cines is faster and more reproducible than manual contour-tracing; however, accurately tracing basal contours remains challenging. Three automated post-processing software packages (Level 1) were compared to manual assessment. Subsequently, automated basal tracings were manually adjusted using a standardized protocol combined with software package-specific relative-to-manual standard error correction (Level 2). All post-processing was performed in 65 healthy subjects. Manual contour-tracing was performed separately from Level 1 and 2 automated analysis. Automated measurements were considered accurate when the difference was equal or less than the maximum manual inter-observer disagreement percentage. Level 1 (2.1 ± 1.0 min) and Level 2 automated (5.2 ± 1.3 min) were faster and more reproducible than manual (21.1 ± 2.9 min) post-processing, the maximum inter-observer disagreement was 6%. Compared to manual, Level 1 automation had wide limits of agreement. The most reliable software package obtained more accurate measurements in Level 2 compared to Level 1 automation: left ventricular end-diastolic volume, 98% and 53%; ejection fraction, 98% and 60%; mass, 70% and 3%; right ventricular end-diastolic volume, 98% and 28%; ejection fraction, 80% and 40%, respectively. Level 1 automated cardiac function post-processing is fast and highly reproducible with varying accuracy. Level 2 automation balances speed and accuracy.

## 1. Introduction

Cardiovascular magnetic resonance (CMR) imaging is the reference standard for non-invasive assessment of ventricular volumes, function, and left ventricular (LV) mass [1]. Conventional post-processing by manually contour-tracing a stack of short-axis slices in end-diastolic and end-systolic phase is time-consuming [2,3]. As an alternative, commercially available artificial intelligence software has the ability to reduce post-processing time while increasing reproducibility [4], and several studies already showed promising results for Level 1 automated assessment [5,6,7,8,9,10]. The major challenge in measurement accuracy is basal tracing; dealing with cardiac through-plane motion combined with the complexity of basal anatomy which also varies between individuals [3,11]. These introduce disagreement with manual reference tracings, leading to different standards being used between sites, especially for the right ventricle (RV) [5,8,12]. Furthermore, Level 1 automated measurements can contain significant vendor-specific relative standard error in all slices [6,8,13].

To address these measurement inaccuracies in commercially available Level 1 automated post-processing solutions, the first phase of the Level 2 automated approach includes standardized manual adjustments of the Level 1 automated basal tracings. In the second phase of Level 2 automation, these results are corrected using the software package-specific relative-to-manual standard errors. Standardization of manual adjustments improves generalizability and reproducibility [14,15,16]. The Society for Cardiovascular Magnetic Resonance (SCMR) published general recommendations for standardized image interpretation of short-axis cines, including statements for identification of the basal slice and inclusion of specific anatomical structures [2]. Detailed operating procedures on handling basal slices with partially visualized LV or RV are nevertheless not available yet.

The first aim was to compare Level 1 automated post-processing of three commercially available software packages to manual contour-tracing on analysis time, reproducibility, and accuracy. The second aim was to assess the added value of the two phases of Level 2 automation; phase 1, manual adjustment of Level 1 generated basal tracings using a standardized contour-tracing protocol incorporating SCMR recommendations, and phase 2, correction of software package-specific relative-to-manual standard errors.

## 2. Materials and Methods

### 2.1. Study Population

**Figure 1:**
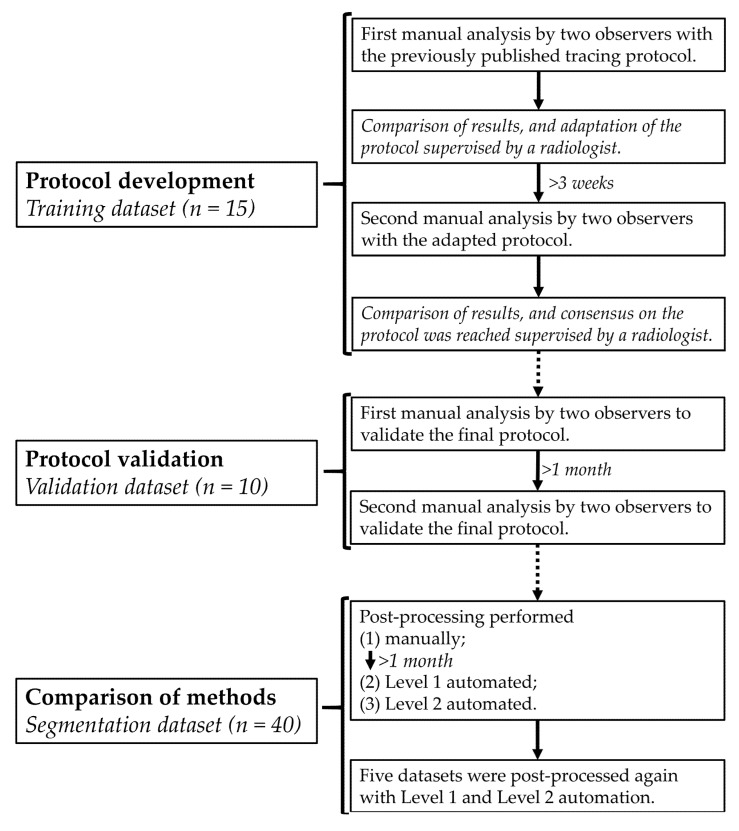
Flowchart of the study design.

This prospective single-center study was conducted in accordance with the Declaration of Helsinki, and the study protocol was approved by the Medical Ethical Committee of the University Medical Center Groningen (no. 2016/476; 19 December 2016). Sixty-five healthy volunteers (mean age, 30 ± 6 years; male sex, 57%) were recruited and signed informed consent prior to inclusion. The included subjects were divided among a training dataset (*n* = 15), validation dataset (*n* = 10) and segmentation dataset (*n* = 40) (study design in Figure 1).

### 2.2. CMR Imaging

All subjects underwent CMR on a 3.0 T scanner (MAGNETOM Prisma, Siemens Healthineers, Erlangen, Germany—software version VE11C) equipped with a 60-element phased-array coil. Electrocardiographically gated balanced steady-state free precession sequences were used to acquire long- and short-axis cines of 25 phases during breath-holds [17]. Long-axis cines were visualized in 4-chamber (4Ch), 2-chamber left ventricle (2ChLV), 2-chamber right ventricle (2ChRV) and outflow tract views. Short-axis cines were acquired from the atria to the ventricular apex with 6 mm slice thickness and 4 mm interslice gap [17]. All images were anonymized and exported directly from the scanner for offline analysis.

### 2.3. Contour-Tracing Protocol

Two observers (a PhD student and a radiology resident, designated first and second), independently evaluated the previously published 1.5 T short-axis contour-tracing protocol using the training dataset (Figure 1) [18]. Manual contour-tracing was performed on a clinical workstation with contour-tracing software cvi42 (v.5.10.1, Circle Cardiovascular Imaging Inc., Calgary, AB, Canada), further referred to as vendor 1, enabling simultaneous comparison of long- and short-axis images. After both observers analyzed the training dataset once, the tracing protocol was adjusted to improve reproducibility in a consensus meeting with an experienced cardiovascular radiologist. Adjustments included adaptation of rules how to in- or exclude the basal tracings, control mechanisms between LV and RV stroke volume and between end-diastolic and end-systolic LV mass, and CMR scan parameters, most importantly slice and interslice gap thickness, and number of phases [18]. After 3 weeks, both observers repeated the manual analysis of the training dataset using the adapted tracing protocol, after which consensus was reached on the final protocol between the above mentioned three observers.

The contour-tracing protocol with detailed textual and visual instructions is provided in Supplementary Material File S1. In brief, the cardiac phases with largest and smallest blood pool in the midventricular short-axis slice were defined as end-diastolic and end-systolic phase, respectively. In these two cardiac phases in all short-axis slices, endocardial contours were traced surrounding the LV and RV blood pool to assess volumes, and additionally epicardial contours surrounding the LV wall for LV mass calculation. Papillary muscles and trabecular tissue were included in the blood pool.

The two observers independently manually validated the contour-tracing protocol using the blinded validation dataset (Figure 1). After one month, both observers retraced the validation dataset to measure reproducibility.

### 2.4. Post-Processing Methods

The segmentation dataset was used to compare Level 1 and phase 1 of Level 2 automated results with gold standard manual contour-tracing results (Figure 1). Analysis time of all measurements was noted. Manual contour-tracing was performed by the first observer with vendor 1, the second observer checked all manual contours for adherence to the final tracing protocol.

Level 1 automated post-processing started one month after finishing manual contour-tracing. The first observer assessed three commonly used commercially available software packages; vendor 1, vendor 2 (Qmass MR, v.8.1, Medis Medical Imaging Systems, Leiden, The Netherlands) and vendor 3 (syngo.via, v.VB30A, Siemens Healthineers, Erlangen, Germany). The hardware specifications met the criteria as recommended by the vendors. All Level 1 automated contour-tracing was performed on short-axis cines exactly adhering to the instructions as provided in their respective user manuals. In all three software packages, the LV range was manually defined by selecting the mitral valve plane and apex on the 4Ch view. As vendor 1 allowed the use of multiple long-axis cines to define ventricular ranges, the LV range was also defined on the 2ChLV, and the RV range was defined on the 4Ch and 2ChRV. In vendor 2 it was not possible to define the RV range, and vendor 3 did not provide automated RV segmentation and was therefore excluded for RV evaluation.

Level 1 automated post-processing was performed with at least two weeks between vendors, and all subjects were post-processed in random order. None of the vendors used machine learning adapting to operator input, and therefore Level 1 automated contour-tracings were not affected by learning from user input. The first five datasets were automatically post-processed again for reproducibility assessment, and since this reproducibility proved to be nearly perfect, the number of datasets was not enlarged.

Phase 1 of Level 2 automated post-processing was performed directly after Level 1 automated assessment was completed. The analysis included manually adjusting the Level 1 automated contours of the most basal slice containing both LV and RV, and the even more basal slice, if only LV or RV remained visible, using the final tracing protocol. Subsequently, adherence to this protocol was checked by the second observer. Phase 1 of Level 2 automated procedure was repeated in the first five datasets, and this number proved to be sufficient to reliably assess post-processing reproducibility.

### 2.5. Statistical Analysis

The statistical analysis was performed using SPSS (v.24, Statistical Package for the Social Sciences, International Business Machines, Armonk, NY, USA). A *p*-value < 0.05 was considered significant. Analysis time was noted as mean ± standard deviation (SD) and analyzed using the analysis of variances with post-hoc tests. Reproducibility was analyzed using the intraclass correlation coefficient (ICC) and Bland-Altman statistics, noted as mean difference (±1.96 SD) (95% limits of agreement) [19,20].

Agreement between methods was analyzed using Bland-Altman statistics and linear regression to investigate which part of the variation was removed by adjusting Level 1 automated basal tracings. Furthermore, the ratio between phase 1 of Level 2 automation and manual tracing results was calculated for each cardiac parameter. The median of this ratio was defined as the software package-specific relative-to-manual standard error used for phase 2 of Level 2 automation. Measurements differing equally or less than the maximum inter-observer disagreement percentage of manual tracings were considered accurate.

Based on Koo et al. [20], ICC ≥ 0.75 was used to define good reliability. The ICC between Level 1 automated and manual tracing results was calculated for volumes and mass per vendor. If Level 1 automated results were unreliable, correcting basal slices alone was not expected to gain sufficiently accurate results. The statistical analysis of Level 2 automation was focused on vendors in which all parameters had an ICC ≥ 0.75.

## 3. Results

### 3.1. Analysis Time

**Figure 2:**
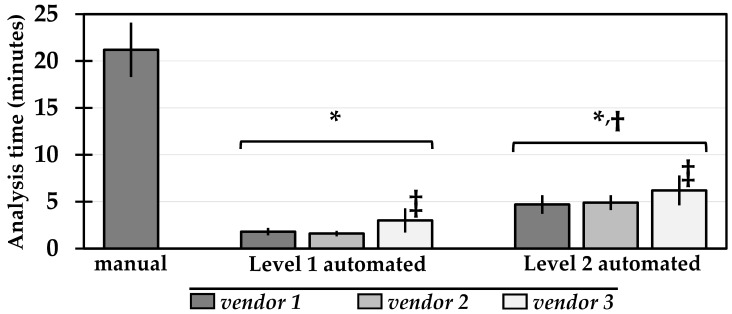
Analysis time (mean ± standard deviation) of each post-processing method. *p* < 0.05 (*) versus manual, (†) versus Level 1 automated, (‡) versus other vendors within the same Level of automation.

The analysis time of manual segmentation (21.1 ± 2.9 min) was significantly longer than Level 1 (2.1 ± 1.0 min) and Level 2 automated (5.2 ± 1.3 min) post-processing (Figure 2).

### 3.2. Reproducibility

The reproducibility of the manually contour-traced validation dataset is shown in Table S1. The intra- and inter-observer agreement were excellent for all parameters (ICC ≥ 0.925).

The intra-observer variability of the LVEF was −0.5% (±1.6%) and the inter-observer variability was −0.4% (±1.5%); for the RVEF, these were −0.2% (±1.2%) and −0.1% (±2.3%), respectively. The maximum inter-observer disagreement was 6%.

The reproducibility of the repeated Level 1 and phase 1 of Level 2 automated measurements is shown in Table S2. The reproducibility of all parameters was close to perfect in both Level 1 (ICC = 0.974–1.000) and phase 1 of Level 2 automated post-processing (ICC = 0.949–1.000), higher than manual tracing. As expected, Level 1 post-processing showed higher reproducibility than phase 1 of Level 2 post-processing.

### 3.3. Accuracy

#### 3.3.1. Level 1 Automation

**Figure 3:**
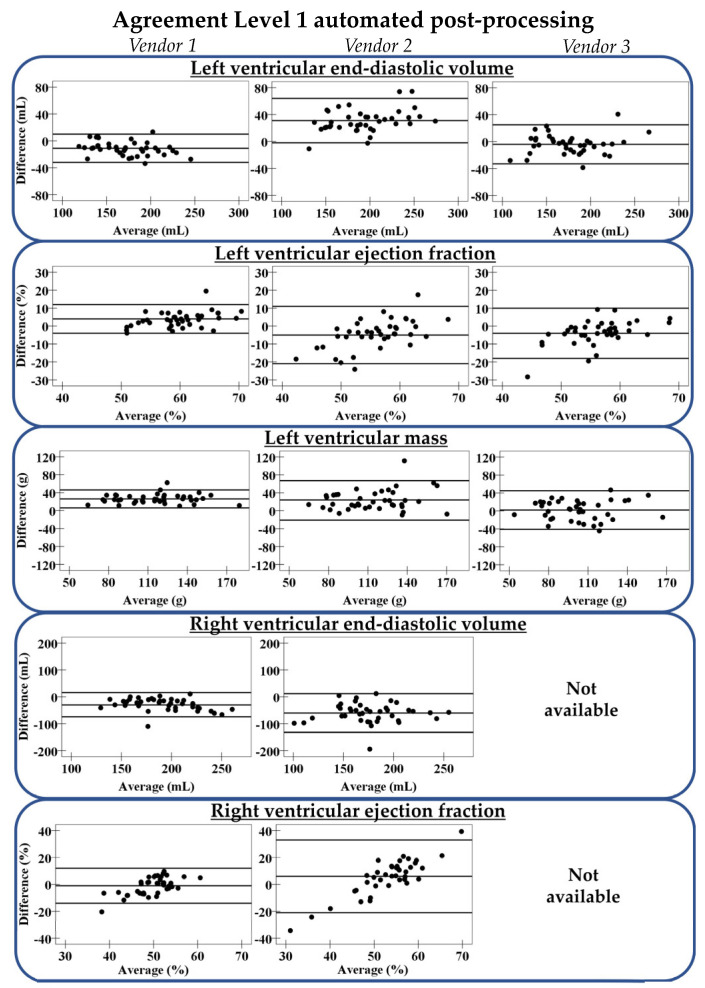
Agreement between Level 1 automation and gold standard manual contour-tracing. The three lines represent mean difference with limits of agreement. Vendor 3 does not support automated right ventricular post-processing.

**Figure 4:**
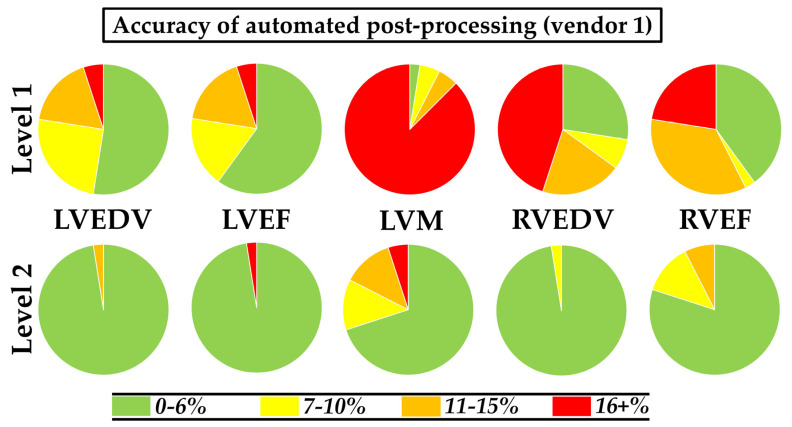
Accuracy of Level 1 and Level 2 automated post-processing results in vendor 1 compared to gold standard manual contour-tracing results. LV: left ventricle; EDV: end-diastolic volume; EF ejection fraction; M mass; RV: right ventricle.

LV parameters differed significantly from the gold standard manual measurements, and this variation was software package-specific (Figure 3, Table S3). Of the three software packages, vendor 1 performed best; the difference in LV end-diastolic volume (EDV) was −11 mL (±21 mL), in LV ejection fraction (EF) 4% (±8%), and in LV mass 26 g (±20 g). RV parameters showed larger differences than LV parameters compared to manual measurements. This variability in vendor 1 led to a small number of Level 1 measurements considered accurate (Figure 4, Table S4).

Despite the differences between vendor 1 and the manual ground truth, the reliability was good for all cardiac parameters (lowest ICC was 0.771, highest ICC was 0.949) (Table S5). In vendor 2 and vendor 3, however, the reliability was moderate for all parameters (ICC ≤ 0.749), except for the LVEDV. Therefore, further evaluation of Level 2 automation focused on vendor 1.

#### 3.3.2. Level 2 Automation

**Figure 5:**
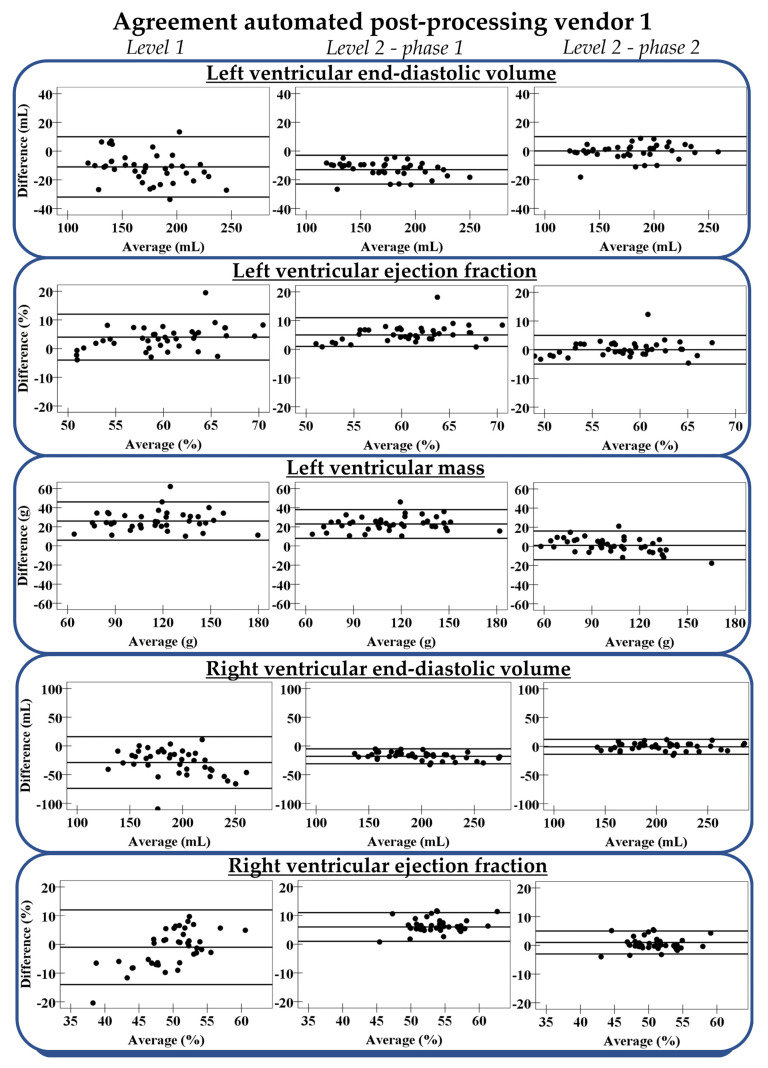
Agreement between different Levels of automated post-processing in vendor 1 and gold standard manual contour-tracing. From left to right: Level 1, Level 2 before correction of the relative-to-manual standard error (phase 1), and Level 2 after correction (phase 2). The three lines represent mean difference with limits of agreement.

The acquired Level 1 automated basal tracings in vendor 1 were manually adjusted using the standardized protocol (phase 1 of Level 2 automation), removing a large part of the variation between automated and manual measurements, especially for RV parameters (Table S3). The R^2^ improved for the LVEDV from 0.91 to 0.98, LVEF from 0.53 to 0.72, LV mass from 0.86 to 0.93, RVEDV from 0.62 to 0.97, and RVEF from 0.17 to 0.63. Corresponding limits of agreement between phase 1 of Level 2 automated and manual results were smaller than those in Level 1 automation (Figure 5). Nevertheless, only a small number of phase 1 of Level 2 automated measurements was considered accurate (Table S4), as the software package-specific relative-to-manual standard error was still present.

The standard error in phase 1 of Level 2 automated measurements is provided in Table S6. The standard error for LVEDV was −7% (interquartile range (IQR); −8%, −6%) and for RVEDV this was −8% (IQR; −11%, −7%), suggesting that volumes were underestimated compared to manual contour-tracing. For the LV mass, this was the opposite as the standard error was positive (21% (IQR; 18%, 29%)). After correcting for these standard errors, most measurements in phase 2 of Level 2 automation were considered accurate: 98% of the LVEDV, 98% of the LVEF, 70% of the LV mass, 98% of the RVEDV, and 80% of the RVEF (Figure 4). Differences between phase 2 of Level 2 automated and manual results are shown in Figure 5.

Level 2 automated results of vendor 2 and vendor 3 are reported in Tables S3–S7. The differences with manual results were larger in vendor 2 and vendor 3 than in vendor 1. After correcting for the relative-to-manual standard errors, the number of accurate measurements in vendor 2 and vendor 3 remained low.

## 4. Discussion

In this study we compared the speed and accuracy of Level 1 automated post-processing to manual contour-tracing. We additionally assessed the benefit of manually adjusting Level 1 automated basal tracings using a standardized protocol (phase 1 of Level 2 automation), combined with correction of the relative-to-manual standard error (phase 2 of Level 2 automation). Level 1 automated post-processing was ten times faster with higher reproducibility compared to manual contour-tracing, however, big differences resulted in few measurements that were considered accurate with large variation between software packages. Vendor 1 demonstrated the most reliable Level 1 automated results, and Level 2 automation showed high accuracy while preserving speed.

Level 1 automated post-processing in all three software packages was quick and highly reproducible, and this could improve clinical workflow as previously reported [8]. However, a large percentage of automated post-processing results was considered inaccurate, as the difference with manual results was higher than 6% (the maximum inter-observer disagreement). Therefore, automated post-processing seems inadequate for implementation in clinical workflow when contours remain unchecked. This supports the SCMR recommendation that the observer must check automated tracings for appropriateness [2].

Literature confirms that basal tracings introduce substantial variation between automated and manual post-processing [3,5,8,10,12], further validating manual adjustments. These adjustments should be standardized into easy to follow tracing instructions which incorporate SCMR recommendations (Supplementary Material File S1) [2,18]. Manual validation of these instructions showed higher intra- and inter-observer reproducibility than previous studies [6,16,21,22,23,24,25], proving it suitable for standardized manual correction of Level 1 automated basal tracings. The lower reproducibility in other studies could be caused by lacking standardization of unambiguous tracing instructions. In the image analysis section some studies referred to the SCMR guidelines [6,22], mentioned the definition of the most basal LV slice [21,22,23,24,25], or described structures that needed to be included in the RV [6,16,21,23,24]. Only Petersen et al. [23] showed similar reproducibility, possibly related to their control mechanisms which included checking equality of end-diastolic and end-systolic LV mass, and LV and RV stroke volumes in absence of significant valvular insufficiency, minimizing discrepancies. The time involved in manual contour-tracing both LV and RV using the standardized instructions (21 min) was representative for clinical routine [3,5], and comparable to previous studies that needed 13 to 14 min for only LV segmentation [26,27].

Vendor 1 demonstrated the most reliable automated post-processing with small variation compared to manual results after phase 1 of Level 2 automation, confirming differences in basal tracings are still the most challenging in automation. The other two vendors showed less reliable Level 1 automated post-processing, and substantial variation remained after adjusting the basal tracings, caused by inaccurate tracings in other slices. Consequently, phase 1 of Level 2 automation is only advantageous in software packages with relatively reliable Level 1 automation.

In all vendors, Level 1 automated contours are consistently traced too narrow or too wide resulting in relative-to-manual standard errors which, if known, can be used to improve accuracy. This correction step was performed during phase 2 of Level 2 automation. In the most reliable package (vendor 1), Level 1 automated volumes were systematically underestimated, and mass overestimated, caused by the endocardial contour being traced comparatively too close to the blood pool in all slices. After correction for this software package-specific relative-to-manual standard error, most measurements were considered accurate. In the other two packages, the calculated relative-to-manual standard errors were imprecise, evidenced by large IQRs, and this was caused by erroneous contour-tracing. In these cases, this final correction step is not beneficial.

In the best performing software package, completed Level 2 automation was still four times faster with higher reproducibility compared to manual, while the variability of volumes and mass remained comparable to literature [6,21,22,23,24]. Interestingly, the variability of LVEF and especially RVEF in vendor 1 was lower than previously reported [6,21,22,24]. This demonstrates that the Level 2 automation approach can be an effective trade-off in software packages with reasonably well Level 1 automated post-processing.

There were several limitations in this study. First, we investigated three mainstream commercially available clinical software packages to perform automated post-processing where more software packages are available. However, we feel that the included software packages are representative of the field, as they are widely used in clinical assessment. Second, we performed gold standard manual contour-tracing on vendor 1 only. Nevertheless, this should not bias the results, as the included software packages ought to provide similar results as demonstrated previously [28]. Third, the segmentation dataset was post-processed multiple times which could induce some memory bias; however, this effect was minimized because of the time between measurements. For Level 1 automation, this bias was also irrelevant as user input was only required to define the ventricular range on long-axis cines, a step not applicable for manual contour-tracing. Fourth, we exclusively imaged healthy subjects in a relatively small group. Reproducibility of presented findings therefore need to be confirmed in a larger patient population with altered chamber geometry.

## 5. Conclusions

In the three studied vendors, Level 1 automated post-processing of cardiac volumes, function, and LV mass showed high reproducibility and speed 10-times faster than manual contour-tracing, however, when left unchecked differing degrees of variation made them inadequate for clinical use. Level 2 automation combines protocolized manual adjustments of Level 1 automated basal tracings with software package-specific relative-to-manual standard error correction while still four-times faster than manual assessment. In a reliable software package, Level 2 automation could be an effective approach to balance speed and accuracy in clinical workflow.

## Data Availability

The data presented in this study are available on request from the corresponding author. The data are not publicly available due to privacy.

## Supplementary Materials

The following are available online at www.mdpi.com/article/10.3390/diagnostics11101758/s1, File S1: contour-tracing protocol. Table S1: Manual reproducibility, Table S2: Automated reproducibility, Table S3: Comparison between methods, Table S4: Accuracy of automated post-processing, Table S5: Intraclass correlation coefficient between automated and manual, Table S6: Software package-specific relative-to-manual standard error, Table S7: Differences between phase 2 of Level 2 automation and manual.
