# Supplementary material for: Balancing Speed and Accuracy in Cardiac Magnetic Resonance Function Post-Processing: Comparing 2 Levels of Automation in 3 Vendors to Manual Assessment
Source: Diagnostics (Basel). 2021 Sep 24;11(10):1758. doi: 10.3390/diagnostics11101758 (PMC8534796; doi:10.3390/diagnostics11101758)
Supplement: Supplementary file 1 [file diagnostics-11-01758-s001.zip › Diagnostics - Snel et al. (2021) - Supplementary File 1 Tracing protocol.pdf]

Contour tracing protocol

General

Accurate acquisition in cardiovascular magnetic resonance is of major importance to be able to follow post-processing contour-tracing protocols and reproducibly quantify cardiac morphology and function. Localization of short-axis slices can be visualized on long-axis cines. Acquisition of three long-axis cines for both left ventricle (LV) (4-chamber (4Ch), 2-chamber left ventricle (2ChLV) and left ventricular outflow tract (LVOT)) and right ventricle (RV) (4Ch, 2-chamber right ventricle (2ChRV) and right ventricular outflow tract (RVOT)) provides sufficient spatial information for correct position localization of the short-axis slices [1,2]. In clinical practice, however, RV long-axis cines besides the 4Ch are not always acquired, and therefore RV post-processing guidelines should also be applicable on short-axis images and 4Ch only. Accurate consistent angulation of short-axis slices on the 4Ch and 2ChLV in end-diastole is necessary to acquire a stack of short-axis slices parallel to the mitral valve from base towards apex [1,2].

The adapted protocol is suited to be used with slice thicknesses of 6-8 mm and interslice gaps of 2-4 mm to equal 10 mm [1,2]. The phases at midventricular slice with the largest and smallest LV blood volume are selected as the end-diastolic and end-systolic phase, respectively. The endocardial contours are traced in the end-diastolic and end-systolic phase of both LV and RV to enable calculation of blood volumes using Simpson's rule. The calculated blood volumes are used to estimate ejection fractions, stroke volumes and cardiac output. In addition, epicardial contours surrounding the LV wall are traced for LV mass assessment. Due to longitudinal ventricular contraction during systole, contour drawing in the most basal slice can differ between the end-diastolic and end-systolic phase (Fig. 1a) [3,4].

Control mechanisms on the acquired data should be included, if possible, to increase the accuracy of cardiac morphology and function assessment. In absence of shunts or valvular regurgitation, LV and RV stroke volumes should be equal [4]. End-diastolic and end-systolic LV mass should also be equal [5]. Differences in either stroke volumes or mass raise suspicion and suggest tracing errors. Although no criteria are available to what degree

differences remain acceptable, we suggest to critically revise the traced contours when differences exceed 5%.

Left ventricle

The endocardial contours are traced on all slices containing myocardium starting at the most basal slice. The observer confirms the presence of myocardium on long-axis views (4Ch and 2ChLV). The endocardial contours are traced surrounding the entire blood pool including the LVOT (Fig. 1b). Papillary muscles and trabecular tissue are also included in the blood pool volume.

The epicardial contours are traced in each slice surrounding the myocardium. In order to include myocardium, the myocardium should also be visible in the following phase. According to the guideline of the Society of Cardiovascular Magnetic Resonance (SCMR) [4] and the previously published protocol [3], blood volume in the basal slice with less than 50% myocardial circumference is excluded. In basal slices with in between 50% and 100% myocardial circumference and not entirely LV as confirmed by the 4Ch, the blood pool not surrounded by myocardium is excluded with a straight line, traced from where the myocardial circumference ends (Fig. 1c). Inclusion of partial LV in the most basal slice was not mentioned in the SCMR, however, we deviate to avoid overestimation of the LV by excluding atrial blood pool. Observers should be careful to not exclude the LVOT when excluding blood pool without myocardial circumference, and this can be confirmed on the LVOT view (Fig. 1b). In basal slices with a small crescent of lateral myocardium without visible LV blood pool, the epicardial contour should be traced without endocardial contour. The apical slice can be traced similarly to the basal slice if only myocardium is visible [4].

Right ventricle

The RV endocardial contours are traced in each slice that contains RV blood volume if the blood volume remains visible in the following two phases. Moderator band and papillary muscles are included in the blood pool volume. The RV endocontour is traced inside the dark line of artifacts bordering the RV blood pool, and so excluding the ventricular wall.

In order to include the RVOT into the blood pool, the short-axis images should be correlated with the RVOT and 2ChRV view if these cines are available. The pulmonary valve has to be traced in basal slices if there is bulging visible in the short-axis cines, i.e. the pulmonary valve leaflets, within the phase of interest or within two following phases. The pulmonary valve can be traced twice at maximum using a straight line from the inferior side of the bulge on the lateral wall towards the inferior side of the bulge on the medial wall. Without clear visualization of the bulge on the medial wall, the straight line can be traced towards the aorta. Blood pool above the pulmonary valve is consequently excluded from the RV blood volume (Fig. 1d).

The tricuspid valve can be traced in the basal slice. The observer can visualize the presence of the tricuspid valve on the 4Ch and, if available, 2ChRV. The atrium can be excluded by tracing the tricuspid valve between atrium and ventricle, if the valve is clearly visible. Occasionally, the course of the tricuspid valve is not clearly visible in the phase of interest; it is then advised to trace the valve as a straight line from the lateral RV border to the medial border, in the direction of the aortic valve (Fig. 1d). Tracing of straight lines is less dependent on observer interpretation and therefore increasing reproducibility.

References

1. Kramer, C.M.; Barkhausen, J.; Bucciarelli-Ducci, C.; Flamm, S.D.; Kim, R.J.; Nagel, E. Standardized cardiovascular magnetic resonance (CMR) protocols 2020 update. *J. Cardiovasc. Magn. Reson.* **2020**, *22*, 17, doi:10.1186/1532-429X-15-91.

2. Greenwood, J.P.; Ripley, D.P. Components of CMR protocols. In *Cardiovascular MR Manual*; Springer, 2015; pp. 195–233 ISBN 9783319209395.

3. Prakken, N.H.; Velthuis, B.K.; Vonken, E.-J.J.; Mali, W.P.; Cramer, M.-J.J. Cardiac MRI: Standardized Right and Left Ventricular Quantification by Briefly Coaching Inexperienced Personnel. *Open Magn. Reson. J.* **2008**, *1*, 104–111, doi:10.2174/1874769800801010104.

4. Schulz-Menger, J.; Bluemke, D.A.; Bremerich, J.; Flamm, S.D.; Fogel, M.A.; Friedrich, M.G.; Kim, R.J.; von Knobelsdorff-Brenkenhoff, F.; Kramer, C.M.; Pennell, D.J.; et al. Standardized image interpretation and post-processing in cardiovascular magnetic resonance - 2020 update. Society for Cardiovascular Magnetic Resonance (SCMR): Board of Trustees Task Force on Standardized Post-Processing. *J. Cardiovasc. Magn. Reson.* **2020**, *22*, 19, doi:10.1186/s12968-020-00610-6.

5. Petersen, S.E.; Aung, N.; Sanghvi, M.M.; Zemrak, F.; Fung, K.; Paiva, J.M.; Francis, J.M.; Khanji, M.Y.; Lukaschuk, E.; Lee, A.M.; et al. Reference ranges for cardiac structure and function using cardiovascular magnetic resonance (CMR) in Caucasians from the UK Biobank population cohort. *J. Cardiovasc. Magn. Reson.* **2017**, *19*, 18, doi:10.1186/s12968-017-0327-9.

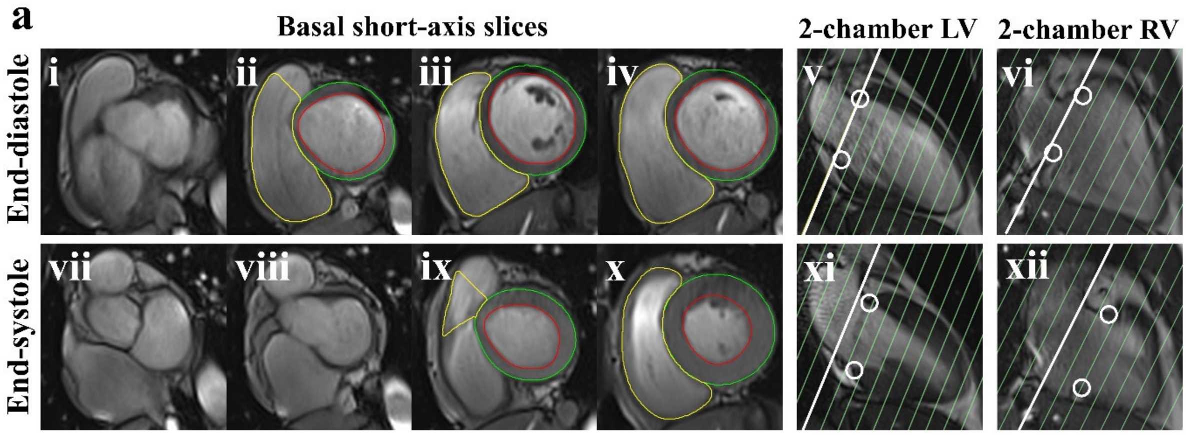

Figure S1a. End-diastolic (*i-iv*) and end-systolic phase (*vii-x*) of basal short-axis slices with corresponding left ventricular (LV) and right ventricular (RV) long-axis images. In the long-axis images, the white line marks the most basal slice and the white circles mark the mitral valve (*v+xi*) and tricuspid valve (*vi+xii*). The most basal slice containing LV and RV differs between end-diastolic and end-systolic phase caused by longitudinal ventricular contraction.

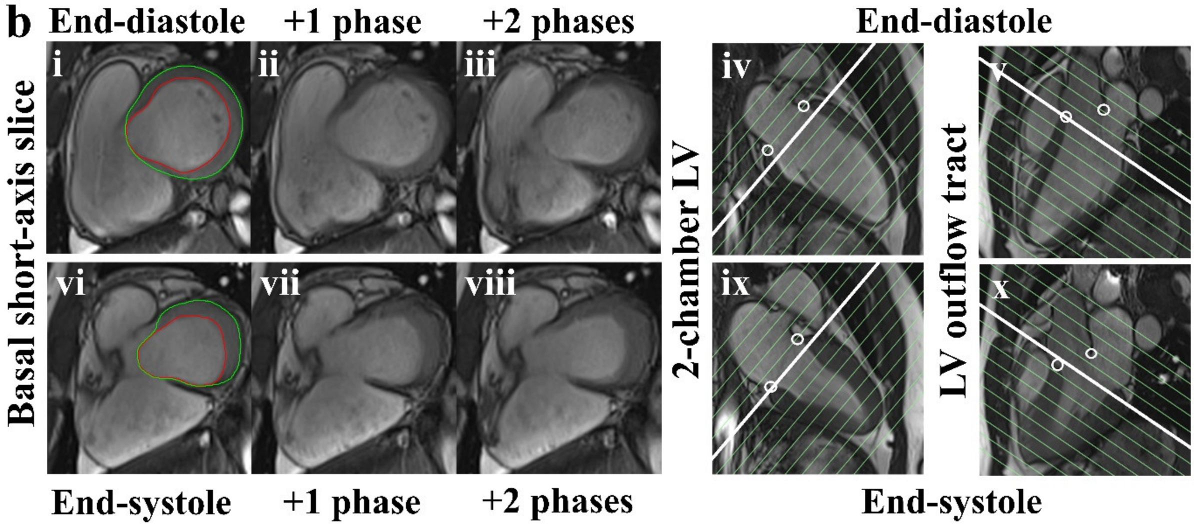

Figure S1b. End-diastolic (*i*) and end-systolic phase (*vi*) with two following phases (*ii*+*iii* and *vii*+*viii*, respectively) of the most basal short-axis slice with corresponding left ventricular (LV) long-axis images (*iv*+*v* and *ix*+*x*, respectively). Due to longitudinal ventricular contraction, (*i-iii*) and (*vi-viii*) are different short-axis slices. The white circles mark the mitral valve (*iv*+*ix*) and aortic valve (*v*+*x*). The blood pool that is not surrounded by myocardium (*i*+*vi*) is the LV outflow tract as confirmed on long-axis images (*v*+*x*) and therefore included in the LV blood pool by matching the endocardial and epicardial contour.

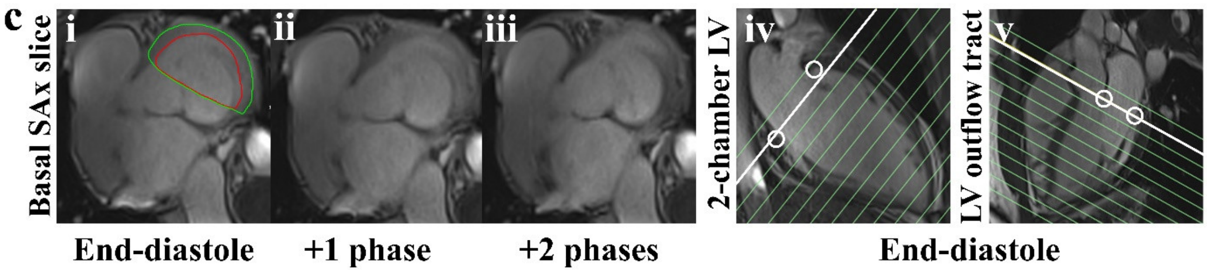

Figure S1c. Basal short-axis (SAx) slice (*i-iii*) with corresponding left ventricular (LV) long-axis images (*iv+v*). The white circles mark the mitral valve. The blood pool without myocardial circumference (*i*) is excluded with a straight line from where the myocardium ends.

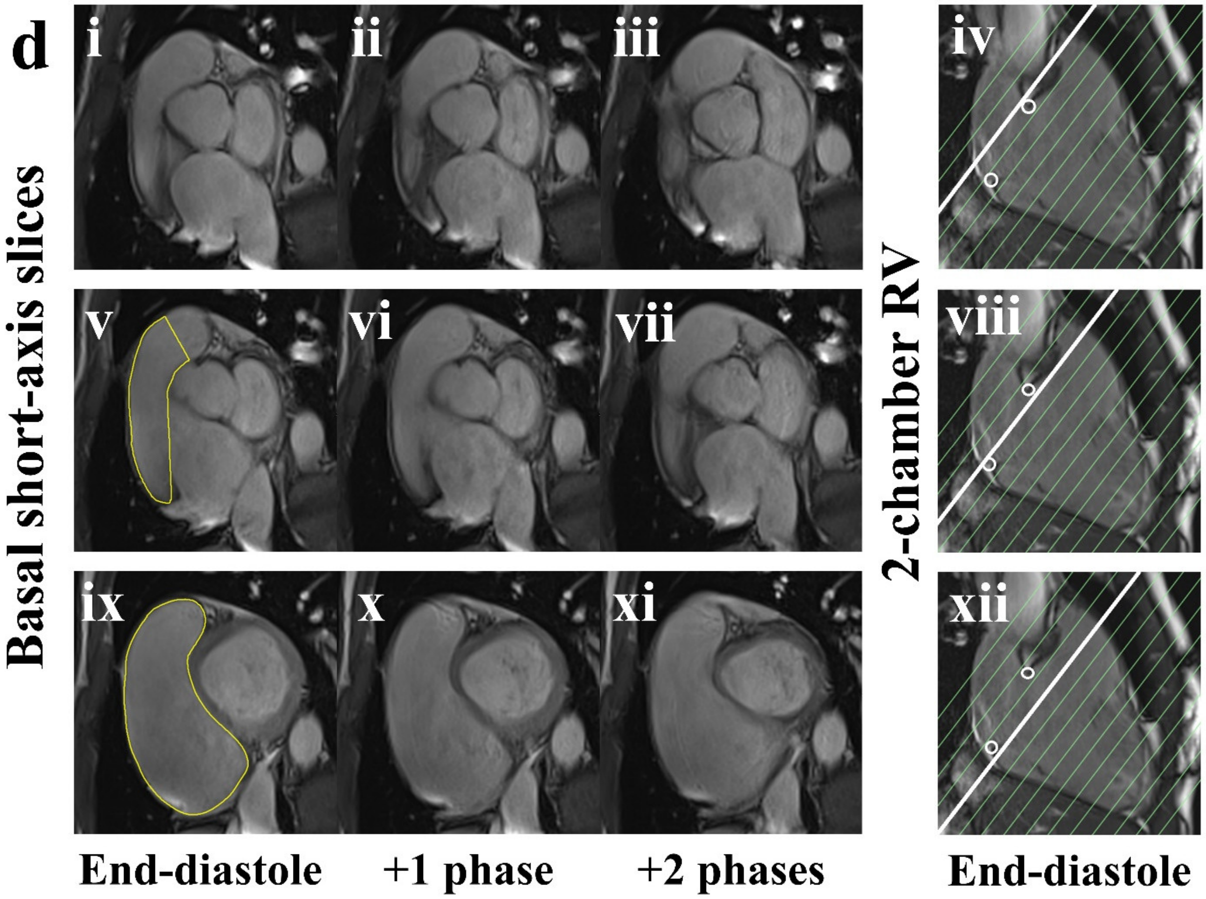

Figure S1d. End-diastolic phase (*i+v+ix*) and two following phases (*ii+iii*, *vi+vii*, *x+xi*, respectively) of three basal short-axis slices with corresponding 2-chamber right ventricle (2ChRV) view (*iv, viii, xii*, respectively). The white circles in the 2ChRV mark the tricuspid valve. In (*i*) there is no blood pool included, because it disappears within two phases. In (*v*) the pulmonary valve is traced with a straight line connecting the visible bulges of the pulmonary valve on the lateral and medial wall (*v-vii*). In (*v*) there is also atrial blood volume, confirmed by (*viii*), and this is excluded with a straight line from the lateral border where the right ventricle ends. In (*ix*) the pulmonary valve is not traced as bulges were not visible (*ix-xi*). The entire blood volume in (*ix*) is included in the endocardial contour as no atrial blood pool is visible in (*ix-xi*), and (*xii*) confirms that the slice is below the tricuspid valve.
