# Supplementary material for: Balancing Speed and Accuracy in Cardiac Magnetic Resonance Function Post-Processing: Comparing 2 Levels of Automation in 3 Vendors to Manual Assessment
Source: Diagnostics (Basel). 2021 Sep 24;11(10):1758. doi: 10.3390/diagnostics11101758 (PMC8534796; doi:10.3390/diagnostics11101758)
Supplement: Supplementary file 1 [file diagnostics-11-01758-s001.zip › Diagnostics - Snel et al. (2021) - Supplementary File 2 Tables.pdf]

Table S1. Reproducibility of the manually contour-traced validation dataset.

	Intra-observer			Inter-observer		
**Parameter**	**Mn-D. (±1.96SD)**	**ICC**	**MD**	**Mn-D. (±1.96SD)**	**ICC**	**MD**
**LVEDV (mL)**	-0.2 (±5.6)	0.988	3	-1.0 (±5.0)	0.989	3
**LVEF (%)**	-0.5 (±1.6)	0.925	4	-0.4 (±1.5)	0.940	4
**LV mass (g)**	0.0 (±5.6)	0.993	5	0.3 (±7.5)	0.989	6
**RVEDV (mL)**	0.6 (±6.5)	0.987	4	0.6 (±8.7)	0.979	4
**RVEF (%)**	-0.2 (±1.2)	0.987	3	-0.1 (±2.3)	0.963	4

Mn-D.: mean difference; SD: standard deviation; ICC: intraclass correlation coefficient; MD: maximum disagreement percentage; LV: left ventricle; EDV: end-diastolic volume; EF: ejection fraction; RV: right ventricle.

Table S2. Reproducibility of repeated Level 1 and phase 1 of Level 2 automated post-processing.

	Vendor 1		Vendor 2		Vendor 3	
	**Mn-D. (±1.96SD)**	**ICC**	**Mn-D. (±1.96SD)**	**ICC**	**Mn-D. (±1.96SD)**	**ICC**
**Parameter**			**Level 1**			
**LVEDV (mL)**	-0.3 (±1.2)	1.000	1.4 (±4.0)	0.999	-0.5 (±2.3)	1.000
**LVEF (%)**	0.0 (±0.0)	1.000	-0.1 (±1.6)	0.974	-0.3 (±0.8)	0.999
**LV mass (g)**	-0.9 (±4.2)	0.998	0.2 (±1.2)	1.000	-0.2 (±0.7)	1.000
**RVEDV (mL)**	-0.5 (±2.0)	1.000	0.0 (±0.0)	1.000	-	-
**RVEF (%)**	0.0 (±0.0)	1.000	0.0 (±0.0)	1.000	-	-
			**Phase 1 of Level 2**			
**LVEDV (mL)**	-1.2 (±1.7)	0.999	0.6 (±2.5)	1.000	-0.4 (±3.0)	0.999
**LVEF (%)**	0.2 (±0.4)	0.999	-0.0 (±0.9)	0.999	-0.0 (±0.3)	1.000
**LV mass (g)**	0.7 (±2.8)	0.999	-0.5 (±2.6)	0.999	-0.5 (±2.8)	0.998
**RVEDV (mL)**	-0.4 (±3.1)	1.000	1.2 (±3.5)	0.999	-	-
**RVEF (%)**	0.6 (±2.0)	0.949	0.3 (±0.6)	0.998	-	-

Mn-D.: mean difference; SD: standard deviation; ICC: intraclass correlation coefficient; LV: left ventricle; EDV: end-diastolic volume; EF: ejection fraction; RV: right ventricle.

Table S3. Comparison of results between Level 1, phase 1 of Level 2 automated, and gold standard manual tracing.

	Vendor 1		Vendor 2		Vendor 3	
	**Mn-D. (±1.96SD)**	**R^2**	**Mn-D. (±1.96SD)**	**R^2**	**Mn-D. (±1.96SD)**	**R^2**
**Parameter**			**Level 1**			
**LVEDV (mL)**	-11 (±21)	0.91	31 (±33)	0.83	-4 (±29)	0.83
**LVEF (%)**	4 (±8)	0.53	-5 (±16)	0.12	-4 (±14)	0.21
**LV mass (g)**	26 (±20)	0.86	24 (±45)	0.46	2 (±43)	0.43
**RVEDV (mL)**	-29 (±45)	0.62	-60 (±72)	0.27	-	-
**RVEF (%)**	-1 (±13)	0.17	6 (±27)	0.02	-	-
			**Phase 1 of Level 2**			
**LVEDV (mL)**	-13 (±10)	0.98	25 (±26)	0.91	-16 (±19)	0.93
**LVEF (%)**	5 (±6)	0.72	-0 (±15)	0.21	2 (±10)	0.41
**LV mass (g)**	23 (±15)	0.93	18 (±32)	0.68	-3 (±41)	0.45
**RVEDV (mL)**	-18 (±13)	0.97	-31 (±43)	0.66	-	-
**RVEF (%)**	6 (±5)	0.63	4 (±16)	0.01	-	-

Mn-D.: mean difference; SD: standard deviation; LV: left ventricle; EDV: end-diastolic volume; EF: ejection fraction; RV: right ventricle.

Table S4. Number of measurements considered accurate with Level 1 and phases 1 and 2 of Level 2 automated post-processing.

	Vendor 1		Vendor 2		Vendor 3	
**Parameter**	**Level 1**					
**LVEDV**	21 (53%)		2 (5%)		26 (65%)	
**LVEF**	24 (60%)		15 (38%)		21 (53%)	
**LV mass**	1 (3%)		8 (20%)		8 (20%)	
**RVEDV**	11 (28%)		4 (10%)		-	
**RVEF**	16 (40%)		8 (20%)		-	
	**Level 2**					
	**Relative-to-manual median standard error correction**					
	**Before**	**After**	**Before**	**After**	**Before**	**After**
**LVEDV**	21 (53%)	39 (98%)	3 (8%)	28 (70%)	11 (28%)	33 (83%)
**LVEF**	14 (35%)	39 (98%)	15 (28%)	17 (43%)	29 (73%)	33 (83%)
**LV mass**	0 (0%)	28 (70%)	10 (25%)	12 (30%)	12 (30%)	15 (38%)
**RVEDV**	12 (30%)	39 (98%)	9 (23%)	19 (48%)	-	-
**RVEF**	3 (8%)	32 (80%)	9 (23%)	17 (43%)	-	-

Data presented as number (percentage). Level 2 automated results are subdivided between before (phase 1) and after (phase 2) software package-specific relative-to-manual median standard error correction. Results were considered accurate when the outcome differed 6% (the maximum interobserver disagreement) or less from the manual result.

LV: left ventricle; EDV: end-diastolic volume; EF: ejection fraction; RV: right ventricle.

Table S5. Intraclass correlation coefficient between Level 1 automated and manual tracing results for volumes and mass.

Parameter	Vendor 1	Vendor 2	Vendor 3
**LVEDV**	0.949	0.899	0.911
**LVESV**	0.874	0.641	0.743
**LV mass**	0.928	0.670	0.657
**RVEDV**	0.771	0.519	-
**RVESV**	0.829	0.300	-

LV: left ventricle; EDV: end-diastolic volume; ESV: end-systolic volume; RV: right ventricle.

Table S6. Software package-specific relative-to-manual standard error in phase 1 of Level 2 automation.

Parameter	Vendor 1	Vendor 2	Vendor 3
**LVEDV**	-7 (-8, -6)	12 (9, 18)	-9 (-11, -7)
**LVEF**	9 (6, 13)	2 (-10, 8)	4 (1, 6)
**LV mass**	21 (18, 29)	16 (7, 34)	3 (-18, 11)
**RVEDV**	-8 (-11, -7)	-14 (-20, -9)	-
**RVEF**	12 (10, 15)	9 (-3, 18)	-

Standard error reported as median percentage (interquartile range).

LV: left ventricle; EDV: end-diastolic volume; EF: ejection fraction; RV: right ventricle.

Table S7. Difference between phase 2 of Level 2 automated and manual results after relative-to-manual standard error correction.

Parameter	Vendor 1<br>Mn-D. (±1.96SD)	Vendor 2<br>Mn-D. (±1.96SD)	Vendor 3<br>Mn-D. (±1.96SD)
**LVEDV (mL)**	0 (±10)	3 (±22)	0 (±22)
**LVEF (%)**	0 (±5)	-1 (±15)	-1 (±9)
**LV mass (g)**	1 (±15)	1 (±30)	-6 (±40)
**RVEDV (mL)**	-1 (±13)	-3 (±45)	-
**RVEF (%)**	1 (±4)	-1 (±15)	-

Mn-D.: mean difference; SD: standard deviation; LV: left ventricle; EDV: end-diastolic volume; EF: ejection fraction; RV: right ventricle.
